# Cellulose Nanocrystal-Based Sulfatase-Responsive Hydrogel for Sustained Celecoxib Release in Ulcerative Colitis Therapy

**DOI:** 10.34133/bmr.0304

**Published:** 2026-02-03

**Authors:** Panalee Pomseethong, Mydhili Govindarasu, Garima Sharma, Yuyuan Guo, Jomon George Joy, Songrae Kim, Seung-Hwan Lee, Jin-Chul Kim

**Affiliations:** ^1^ Department of Biomedical Science and Institute of Bioscience and Biotechnology, Kangwon National University, Chuncheon 24341, Republic of Korea.; ^2^ Institute of Forest Science, Kangwon National University, Chuncheon 24341, Republic of Korea.; ^3^ Metropolitan Seoul Center Korea Basic Science Institute (KBSI), Seoul 02841, Republic of Korea.

## Abstract

Conventional treatments for ulcerative colitis (UC) are often associated with systemic side effects and require frequent dosing due to nonspecific drug distribution. Herein, a hydrogel based on poly(diallyldimethylammonium chloride) (PDADMAC) and cellulose nanocrystals (CNCs) was designed for the oral delivery of celecoxib in the treatment of UC. The hydrogel network incorporated sulfate ester groups, enabling sulfatase enzymes sensitive in the colon. Physiochemical characterization confirmed successful formation of the hydrogel, effective sulfate functionalization, and efficient drug encapsulation. Swelling studies revealed that the hydrogel maintained structural stability under different pH conditions, while in vitro and in vivo experiments demonstrated that drug release was markedly enhanced in the presence of sulfatase. Furthermore, the hydrogel showed improved drug loading efficiency and sustained release behavior. In a dextran sulfate sodium-induced UC mouse model, the celecoxib-loaded PDADMAC-CNC hydrogel effectively alleviated colonic inflammation, preserved colon structure, and reduced pro-inflammatory cytokine levels more markedly than free drug administration. This finding highlights the potential of sulfatase-responsive PDADMAC-CNC hydrogels as a targeted, safe, and effective approach for treating inflammatory bowel diseases, such as UC.

## Introduction

Ulcerative colitis (UC) is one of the 2 main forms of inflammatory bowel disease (IBD), characterized by mucosal inflammation that begins distally and can extend proximally to involve the entire colon, resulting in repeated cycles of relapse and remission [1,2]. The etiology includes genetic risk, environmental factors, immune dysregulation, and gut microbiota imbalances [3,4]. Like many complex diseases, various etiological factors can influence the occurrence of UC and its subsequent course and severity. The most common is bloody diarrhea with abdominal pain, urgency, and tenesmus [5]. In addition to significantly affecting quality of life and work efficiency due to frailty symptoms, UC is also associated with an increased risk of colorectal cancer (CRC) [6]. In the past decades, the incidence rate of UC has been on the rise [7]. The usual treatment methods include medication and surgery but are accompanied by potential side effects or adverse reactions as well as high treatment costs [8,9]. Therefore, the development of new therapeutic strategies is a pressing need.

The development of advanced drug delivery systems has been well-known during the last decade to overcome the limitations of conventional therapies. Especially hydrogels for oral delivery, the 3-dimensional network structures allow them to protect encapsulated drugs from degradation in harsh conditions [10,11]. Moreover, their biocompatibility, porosity, high water content, and controlled release properties make them suitable oral delivery carriers [12,13]. The traditional hydrogels are deficient in response to the local disease environment, causing nonspecific drug release. To address the limitation, stimuli-responsive hydrogels have been designed for drug release in response to specific conditions such as pH, temperature, redox conditions, and enzyme [14,15]. These smart hydrogels enable precise control of drug release, ensuring that the therapeutic agents are released at the target site.

Among various stimuli, enzyme-responsive hydrogels represent a particularly fascinating system due to their ability to regulate the biological environment in the body [16]. Contrary to systems that require external triggers such as light or magnetic fields, enzyme-responsive systems rely on endogenous enzymes, which are naturally present in specific tissues or disease sites [17,18]. In addition, the high selectivity and effectiveness under mild conditions are unique characteristics of enzymes. Several studies have revealed that sulfatases are a specialized class of hydrolase enzymes predominantly found in the colon [19,20]. They catalyze the hydrolysis of sulfate esters from a wide range of substrates [21]. Importantly, because of their localized abundance and functional significance in the colon, sulfatases are excellent biological triggers for designing enzyme-responsive hydrogels for UC therapy. However, despite the increasing attention toward enzyme-responsive systems, hydrogels based on sulfatase activity as the biological trigger remain largely unexplored, particularly for the colon-targeted delivery.

**Fig. 1.**
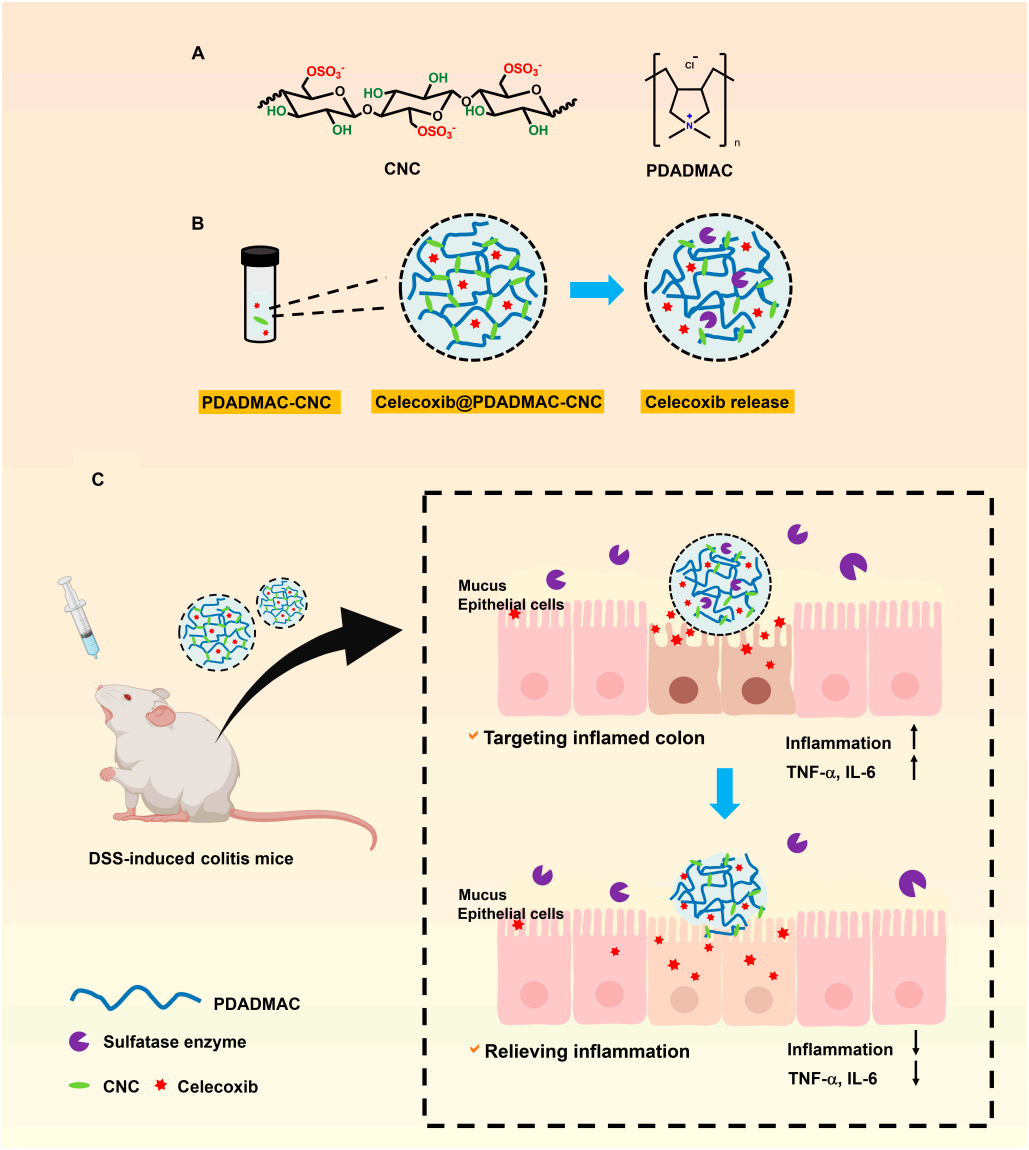
Schematic representation of developed PDADMAC-CNC hydrogel for UC treatment. (A) Chemical structure of PDADMAC and CNC. (B) Illustration of PDADMAC-CNC hydrogel formation and celecoxib-loaded hydrogel. (C) Oral administration of celecoxib-loaded PDADMAC-CNC hydrogel in a DSS-induced UC mouse model.

In this study, we developed a sulfatase-responsive hydrogel platform for targeted therapy of UC, utilizing sulfated cellulose nanocrystal (CNC). CNCs were surface-functionalized via sulfuric acid, rendering them responsive to sulfatase activity and enhancing electrostatic interactions with poly(diallyldimethylammonium chloride) (PDADMAC) to form the hydrogel network (Fig. 1). Additionally, the inherent mucoadhesive properties of CNC enable prolonged retention in the colon [22], thereby facilitating sustained and localized drug delivery. Celecoxib, a selective cyclooxygenase-2 (COX-2) inhibitor, was loaded into the PDADMAC-CNC hydrogel to evaluate therapeutic efficacy in a dextran sulfate sodium (DSS)-induced colitis mouse model. Celecoxib was chosen due to the potent anti-inflammatory activity and reduced gastric toxicity relative to traditional nonselective nonsteroidal anti-inflammatory drugs [23]. Importantly, COX-2 is markedly up-regulated in inflamed colonic tissues during UC, where its overexpression contributes to the production of pro-inflammatory mediators [24], making selective COX-2 inhibition a mechanistically relevant therapeutic approach. Although systemic administration of celecoxib has been associated with cardiovascular risks, these effects are widely recognized to be dose- and exposure-dependent [25]. By achieving localized, colon-specific release, the sulfatase-responsive PDADMAC-CNC hydrogel is designed to minimize systemic absorption and thereby mitigate the potential systemic side effects associated with COX-2 inhibitors. This work presents a novel enzyme-triggered, colon-targeted hydrogel drug delivery system that achieves enhanced therapeutic outcomes in UC.

## Materials and Methods

### Materials

CNC was obtained from Celluforce Inc. (Quebec, Canada). PDADMAC (20 wt % H_2_O), sulfuric acid (95% to 98%), sulfatase from *Helix pomatia*, and DSS (molecular weight, 4 × 10^4^) were purchased from Sigma-Aldrich Co. (St. Louis, MO, USA). Dimethyl sulfoxide anhydrous (DMSO) was obtained from Daejung Chemical & Metals Co. Ltd. (Gyeonggi-do, Korea). DiR (1,10-dioctadecyl-3,3,30,30-tetramethyl indotricarbocyanine iodide) was obtained from Thermo Fisher Scientific. All chemicals were purchased in analytical grade.

### Acid hydrolysis cellulose nanocrystals and characterization

**Fig. 2.**
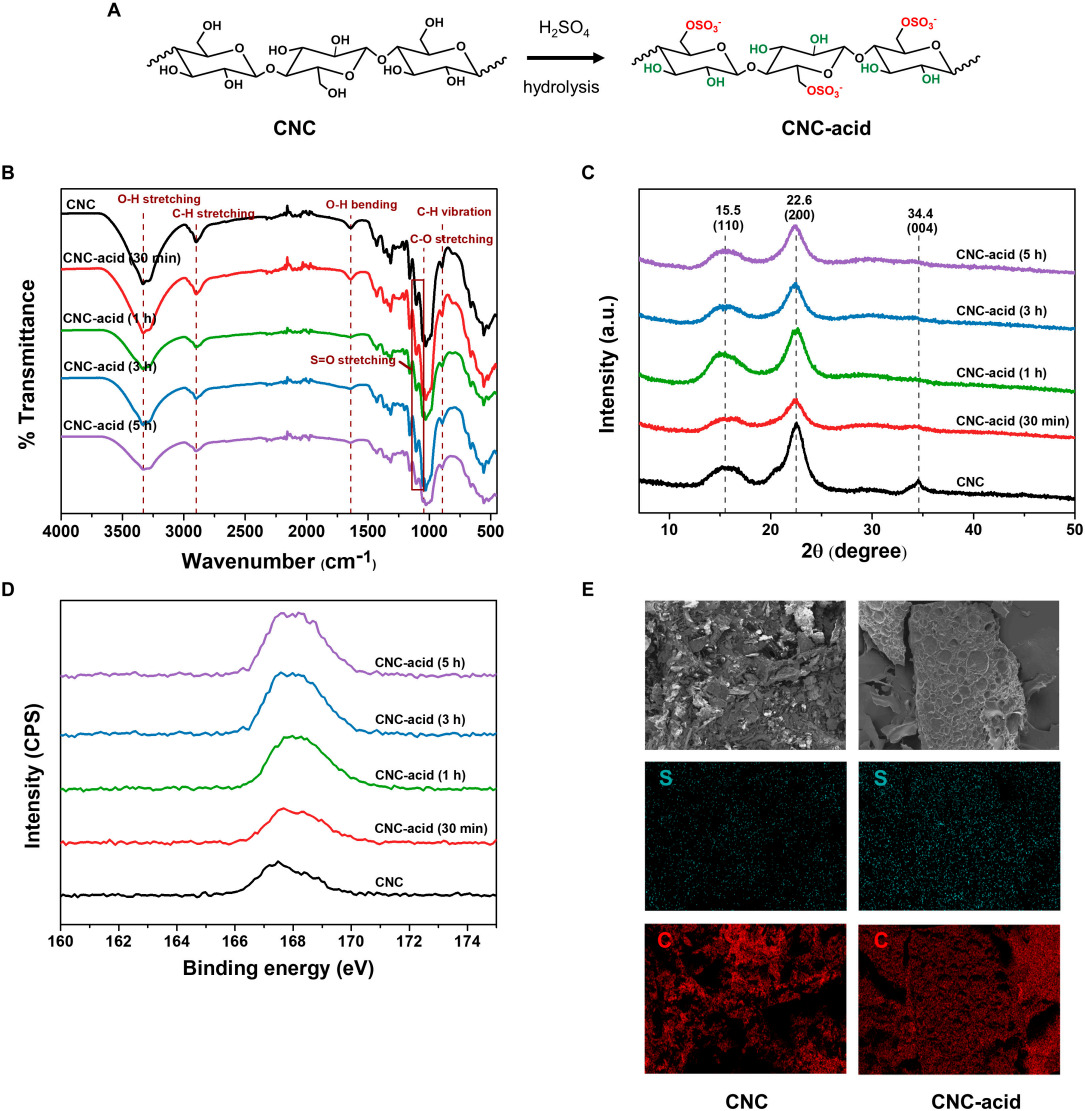
Preparation and characterization of CNC-acid. (A) Schematic representation of CNC-acid by sulfuric acid hydrolysis. (B) FT-IR spectra. (C) XRD patterns. XPS spectra (D) and SEM-EDS analysis of CNC and CNC-acid (E).

A schematic representation of the preparation of acid hydrolysis cellulose nanocrystals (CNC-acid) is illustrated in Fig. 2A. Commercial CNC was acid hydrolyzed in 64% sulfuric acid (H_2_SO_4_) at a temperature of 50 °C in different time treatments (30 min, 1 h, 3 h, and 5 h). The CNC-acid suspension was diluted 10-fold, centrifuged, and followed by washing the sediment with distilled water. The centrifugation and washing were repeated until the pH of the suspension was neutral. The CNC-acid suspension was dialyzed against distilled water for 2 d at room temperature with the medium being exchanged 3 times, and then the suspension was sonicated using tip type for 40 min. The final samples were freeze-dried for characterization and further experiment. The Fourier transform infrared (FT-IR) spectra were taken by FT-IR spectrophotometer (FT-3000-Excalibur, Varian Inc., CA, USA, located at The Central Lab of Kangwon National University), scanning from 4,000 to 400 cm^−1^. CNC and CNC-acid with different time treatments were analyzed. The x-ray diffraction was used to determine the crystallinity of the CNC-acid using an x-ray diffractometer (PANalytical, X’pert-pro MPD, Netherlands) in the range 2θ = 5° to 50°.

The x-ray photoelectron spectroscopy (XPS) signal with elemental analysis was recorded by the x-ray photoelectron spectrophotometer (K, Alpha+, Thermo Scientific, UK). The binding energy of sulfur was observed to analyze the sulfur content. Dynamic light scattering (DLS) equipment was used to study the zeta potential of CNC and CNC-acid. Moreover, scanning electron microscopy (SEM) analysis with elemental mapping was performed using energy-disperse x-ray spectroscopy (EDS) for compositional elements. The synthesized CNC-acid was used for hydrogel preparation.

### Preparation of PDADMAC-CNC hydrogel

To prepare PDADMAC-CNC hydrogels, the CNC was dissolved in distilled water (pH 7.4) and ultrasonicated for 20 min at room temperature to obtain a 5% w/v CNC suspension. Sonication was performed using a tip type with 30% amplitude for 10 s on and then 10 s off. The different concentrations of CNC from 1% to 5% w/v were transferred to vials. Next, PDADMAC was added to the CNC suspension. The PDADMAC-CNC mixtures were mixed well and allowed the gel formation for 4 h at room temperature. The hydrogel samples with different CNC compositions were abbreviated to PDADMAC-CNC(a)% (a was the CNC concentration).

### Characterization of PDADMAC-CNC hydrogel

#### Scanning electron microscope

The morphology of PDADMAC-CNC hydrogels was observed using SEM (JSM-7900F, located at The Central Lab of Kangwon National University). The lyophilized samples were coated with a thin layer of platinum to improve the conductivity and high-resolution structural examination. SEM analysis with elemental mapping was performed using EDS for compositional elements.

#### X-ray diffraction spectroscopy

The x-ray diffraction pattern of the PDADMAC-CNC hydrogel was examined using an x-ray diffractometer. The hydrogels were freeze-dried into powder. Grinding is required to ensure uniform particle size, and then the sample was placed in the diffractometer at the intersection of the x-ray beam and the detector’s path. X-ray radiation was applied to each sample using CuKα (λ = 0.15418 nm). The current and accelerating voltage were 30 mA and 40 kV, respectively. The diffraction angle (2θ) was scanned in the range of 10° to 50°.

### Swelling behavior of PDADMAC-CNC hydrogel

The swelling behavior of the PDADMAC-CNC hydrogel was investigated at 37 °C in different pH values. The hydrogel samples were freeze-dried and weighed as dried weight. Next, dried hydrogels were immersed in distilled water pH 1.4, 6.8, and 7.4 for 2 h. Those pH values refer to simulated gastric fluid (SGF; pH 1.4), simulated small intestinal fluid (SSIS; pH 6.8), and simulated colonic solution (SCS; pH 7.4). The samples were removed from the solutions at a predetermined time point. The weight of the samples was recorded as a wet weight. The swelling behavior was calculated following the equation: Swelling ratio = (wet weight − dried weight)/dried weight × 100%.

### In vitro release profiles of celecoxib drug and DiR from PDADMAC-CNC hydrogel

To determine the release profiles of the PDADMAC-CNC(a%) hydrogel, an anti-inflammatory drug (celecoxib) and DiR dye (drug model) were loaded into the hydrogel. The loaded hydrogel was then left at room temperature to allow gel formation.

The encapsulation efficiency (EE%) and drug loading efficiency (DLE%) of the PDADMAC-CNC(a%) hydrogel were observed to identify the formulation of the hydrogel for the release experiment. The fluorescence intensity versus concentration of celecoxib standard curve was performed (Fig. S1), and the calculation was completed according to the following equation:

$$\begin{array}{c} \text{Encapsulation efficiency}\;\left(\mathrm{EE}\%\right)=\\ \text{Amount of drug loaded}/\text{Amount of drug added}\;\times 100\% \end{array}$$ (1)

$$\begin{array}{c} \text{Drug loading efficiency}\;\left(\mathrm{DLE}\%\right)=\\ \text{Amount of drug loaded}/\text{Total amount of hydrogel}\;\times 100\% \end{array}$$ (2)

The release profiles were studied under different pH conditions to ensure the stability of the hydrogel. The dried hydrogel was immersed in 10 mg/ml aqueous solutions with the following pH values: SGF (pH 1.4), SSIS (pH 6.8), and SCS (pH 7.4). The immersion was observed at 37 °C with 50 rpm shaking speed for 24 h. At predetermined intervals, 1 ml of solution was taken and centrifuged at 10,000 rpm for 2 min. The supernatant was measured using a fluorescence spectrophotometer (F2500, Hitachi, Japan) by analyzing fluorescence intensity at 360 nm with an excitation wavelength of 272 nm. After examination, the solution was transferred back to maintain the volume constant. The amount of celecoxib drug released from the hydrogel was determined on a standard curve.

The release profiles of DiR were determined similarly, except the fluorescence intensity was measured at 760 nm with an excitation wavelength of 754 nm. Likewise, the amount of DiR dye released from the hydrogel was measured by calculating from a standard curve.

For the sulfatase enzyme-responsive release of celecoxib drug and DiR dye from the PDADMAC-CNC(5%) hydrogel, the dried hydrogel was immersed in 10 mg/ml aqueous solutions containing sulfatase enzyme 0, 20, 50, and 100 U/ml at SCS (pH 7.4). The immersion was conducted at 37 °C and 50 rpm shaking speed for 24 h. Experimental procedures for measuring the release profiles were performed as mentioned above. The release amount was assayed by a fluorescence spectrophotometer based on the standard curve. The release experiment was performed 3 times in duplicate, and the mean values are shown.

### In vitro inflammatory response

To evaluate the anti-inflammatory effects of the celecoxib@PDADMAC-CNC hydrogel in the DSS-induced UC model, Caco-2 human colorectal adenocarcinoma cells were pretreated with DSS (3% w/v) [26] for 24 h to mimic the inflammatory microenvironment of UC. Cells were then treated with free celecoxib, free PDADMAC-CNC hydrogel, and celecoxib@PDADMAC-CNC hydrogel. After 24 h, the supernatant of each well was collected, and the level of pro-inflammatory cytokines [tumor necrosis factor-α (TNF-α) and interleukin-6 (IL-6)] was analyzed by enzyme-linked immunosorbent assay (ELISA). Caco-2 cells stimulated by DSS were employed as the positive control, whereas Caco-2 cells not treated with DSS were used as the negative control.

### Animals and DSS-induced UC mouse model

The animal studies were conducted in accordance with the National Research Council’s Guide for the Care and Use of Laboratory Animals, and animal protocols were approved by the Institutional Animal Care and Use Committee (IACUC) of Kangwon National University (KW-240715-3), following Animal Research: Reporting of In Vivo Experiments (ARRIVE) guidelines. BALB/c male mice (6 weeks, approximately 20 g) were used in the study. After 1 week of acclimation to the new environment, mice were randomly divided into control mice [phosphate-buffered saline (PBS) administered; 5 mice] and DSS-administered mice treated with the PDADMAC-CNC hydrogel (50 mg/kg; 5 mice), celecoxib (3 mg/kg; 5 mice), or celecoxcib@PDADMAC-CNC (50 mg/kg, containing 3 mg/kg drug; 5 mice). All mice from the disease groups were induced to develop UC by drinking water containing 3% w/v DSS. After DSS treatment, samples were orally administered to mice for 7 d.

### In vivo biodistribution evaluation

DiR dye was used as a near-infrared fluorescent probe to evaluate the retention of the PDADMAC-CNC hydrogel in the colon. The mice with DSS-induced UC were orally administered free DiR and DiR@PDADMAC-CNC and then subjected to in vivo fluorescence imaging (IVIS) at predetermined intervals (0, 2, 4, and 6 h). Mice were euthanized at 6 h after administration, and colon, heart, lung liver, kidney, and spleen were taken. The regional fluorescence intensities were analyzed using the software of a living imaging system.

### Evaluation of therapeutic effect on colitis mice

During sample oral administration, the body weight of mice and disease activity index (DAI) score were measured and recorded. The criteria of DAI score to evaluate disease activity severity were based on weight loss (%), stool consistency, and fecal bleeding [27]. After 7 d of drug administration, mice were euthanized under CO_2_ and organs were collected. The colon length and spleen weight were determined. To evaluate systemic inflammation and immune responses in DSS-induced mice, cytokine testing from blood was studied. Blood was collected from the vein in eye socket, then the serum was centrifuged at 4,000 rpm for 10 min, and the supernatant was collected to measure serum TNF-α and IL-6 level.

### Hematoxylin and eosin and immunostaining

The colon tissues were harvested from the mice at the end point of the experiment. The colon tissues were then fixed in 10% neutral-buffered formalin for 24 h, followed by dehydration and embedding in paraffin. Sections of 5 μm were prepared from the paraffin-embedded tissue blocks using a microtome and mounted on New Silane III Slide Glass for histological examination. For all the staining, the paraffin sections were deparaffinized in the histoclear solution and rehydrated through a graded ethanol series. For hematoxylin and eosin (H&E) staining, the slides were stained with Mayer’s hematoxylin, rinsed in tap water, and counterstained with eosin. The slides were then dehydrated in graded ethanol series, cleared in the histoclear solution, and mounted with coverslips using distrene plasticizer xylene (DPX) mounting medium. H&E staining was used to evaluate tissue morphology and integrity.

For immunohistochemical staining, the rehydrated tissue sections were subjected to antigen retrieval and then separately incubated overnight with occludin, claudin-5, or ZO-1 primary antibodies at 4 °C. The occludin and claudin-5 slides were then visualized with horseradish peroxidase (HRP)/3,3'-diaminobenzidine (DAB) avidin–biotin complex (ABC) detection kit (Abcam). The ZO-1 slides were treated with HRP-conjugated secondary antibody and visualized with DAB substrate with nickel enhancement (Vector Labs). After all the immunostainings, the tissues were counterstained with hematoxylin, dehydrated in graded ethanol series, cleared in the histoclear solution, and mounted with coverslips using DPX mounting medium. The slides were visualized using Carl Zeiss (Digital Slide Scanner).

### Statistical analysis

All experiments were performed at least 3 times, and the results are presented as mean ± standard deviation (SD) with 95% confidence intervals. The statistical analysis was performed by using Origin Pro 9.0 and GraphPad Prism version 8. Differences between groups were evaluated using one-way analysis of variance (ANOVA) followed by Tukey’s multiple comparison test. ^ns^*P* > 0.05 was considered as nonsignificant, *P < 0.05 was considered as significant, **P < 0.01 was considered as very significant, and ***P < 0.001 was considered as extremely significant.

## Results and Discussion

### CNC-acid and characterization

Figure 2B shows the FT-IR spectrum of commercial CNC and CNC-acid in different treatment times (30 min, 1 h, 3 h, and 5 h). The obtained spectra demonstrated characteristic peaks of CNC, and the signal at position 3,330 cm^−1^ corresponded to O–H stretching, indicating hydrogen bonding among the hydroxyl group. The signal at 2,899 cm^−1^ represents the C–H stretching, and the peak at 1,160 cm^−1^ is attributed to C–O–C stretching, which indicates the β-(1→4) glyosidic linkages in cellulose. The signal at 1,052 cm^−1^ corresponded to C–O stretching in primary alcohol groups. The signal appeared around 1,029 cm^−1^, which was attributable to the stretching of sulfate ester groups. Moreover, the reduction of the O–H stretching signal in the spectrum of CNC-acid was found, possibly due to the effect of the acid hydrolysis treatment [28]. During the acid hydrolysis, the hydroxyl groups on the CNC surface could be replaced by sulfate groups through the sulfation process, which can decrease the number of free hydroxyl groups and result in the decrease of the O–H stretching signal. This result can confirm that sulfuric acid treatment modified the CNC by inducing more sulfate ester groups.

The x-ray diffraction spectroscopy (XRD) patterns of commercial CNC and CNC-acid are shown in Fig. 2C. All the samples exhibited diffraction peaks at 15.5°, 22.6°, and 34.6°, corresponding to the (110), (200), and (004) crystal planes of cellulose I, respectively [29]. These characteristic peaks confirm that the CNCs retain the cellulose I crystalline structure after sulfuric hydrolysis. The preservation of these diffraction peaks suggests that the acid hydrolysis process removed the amorphous regions and preserved the organized crystalline domains, as evidenced by the clear and sharp diffraction peaks observed in samples [30].

Figure 2D shows the XPS spectra of the S2p peak for commercial CNC and CNC-acid with atomic percentage analysis. The results showed that after the acid hydrolysis, the sulfur 2p signal intensity was gradually increased at binding energy 167.5 eV. The atomic content of all samples was determined from the intensities of the sulfur signal peak, as shown in Table 1. The sulfur content of CNC and CNC-acid (30 min, 1 h, 3 h, and 5 h) increased from 0.51% to 0.63%, 0.88%, 0.81%, and 0.94%, respectively. These results confirm that the sulfuric acid hydrolysis process introduces more sulfate ester groups on the CNC surface as an increased percentage of sulfur.

**Table 1.** The zeta potential of CNC and CNC-acid

Samples	Zeta potential (mV)
CNC	−20.10 ± 0.92
CNC-acid (30 min)	−30.40 ± 0.32
CNC-acid (1 h)	−34.77 ± 0.62
CNC-acid (3 h)	−34.59 ± 0.77
CNC-acid (5 h)	−35.62 ± 0.64

According to the DLS results, the mean zeta potentials of commercial CNC and CNC-acid (30 min, 1 h, 3 h, and 5 h) were −20.10, −30.40, −34.77, −34.59, and −35.62 mV, respectively (Table 1). This is attributted to the presence of sulfate ester groups on the CNC surface [31]. These sulfate groups are produced when sulfuric acid and hydroxyl groups of CNC undergo an esterification process during hydrolysis, resulting in higher density of negatively charged functional groups. As a result, CNC-acid exhibits more negative zeta potential compared to commercial CNC. The increased negative charge enhances electrostatic repulsion between CNC particles, improving the colloidal stability in aqueous suspensions and preventing aggregation, which is crucial for application in drug delivery.

Moreover, the SEM-EDS analysis of CNC and CNC-acid was studied (Fig. 2E). As shown in elemental mapping, the distribution of sulfur (S) content of CNC-acid was higher than that of CNC because the acid hydrolysis process introduced more sulfate ester groups onto the surface of CNC, which increased the S signal detected by EDS. In contrast, the carbon (C) intensity showed no significant difference, as the cellulose structure is primarily carbon-based and remained unchanged by the surface modification.

### Preparation of PDADMAC-CNC hydrogel

**Fig. 3.**
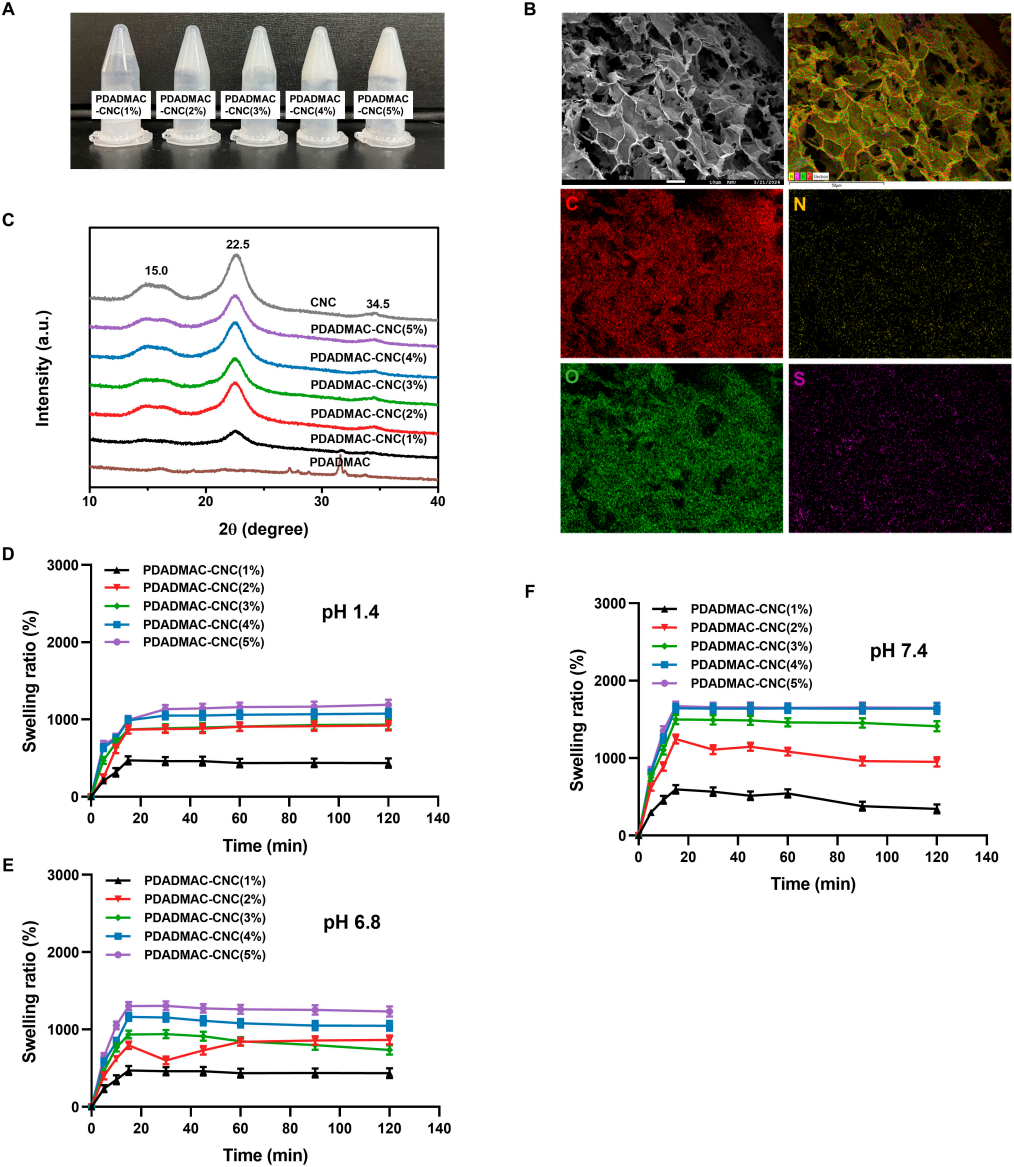
Characterization of PDADMAC-CNC hydrogel. (A) Photographs of PDADMAC-CNC hydrogel (1% to 5% w/v). (B) EDS analysis of C, N, O, and S. XRD patterns (C) and swelling ratio at pH 1.4 (D), 6.8 (E), and 7.4 (F) of PDADMAC-CNC hydrogel.

The photographs of the PDADMAC-CNC hydrogel with different CNC content are shown in Fig. 3A. According to the result, as CNC concentration increased from 1% to 5% w/v, hydrogel stability increased. In the hydrogel matrix, CNC functions as a reinforcing agent. At higher concentrations, CNC provides a more extensive network, enhancing the mechanical strength and rigidity of the hydrogel. Moreover, CNC can participate in the physical crosslinking through hydrogen bonding and electrostatic interactions with PDADMAC. The electrostatic interaction between the positively charged PDADMAC and the negatively charged sulfate ester groups of CNC contributed to the hydrogel formation. Higher CNC content enhances the charge interactions, leading to more hydrogel stability.

### Characterization of PDADMAC-CNC hydrogel

To study the morphological structure of the hydrogel, the freeze-dried PDADMAC-CNC hydrogel was characterized by SEM and EDS mapping (Fig. 3B). The SEM image exhibited an interconnected porous structure, essential for swelling behavior and drug-loading capabilities of the hydrogel. The well-distributed networks suggest efficient crosslinking between PDADMAC and CNC. Drugs can be maintained within the hydrogel and gradually released under responsive conditions for prolonged release and drug encapsulation. Additionally, the EDS mapping results further support the structural observation by identifying the elemental composition of the hydrogel. The elemental mapping revealed the presence of carbon (C) and oxygen (O), the main components of CNC and PDADMAC polymer. The presence of nitrogen (N) was part of the PDADMAC component contained in the quaternary group. The uniform distribution of nitrogen throughout the hydrogel suggested that PDADMAC was well-mixed with the CNC, confirming successful homogeneity of the hydrogel network. Furthermore, the sulfur (S) signal in EDS mapping verified the existence of sulfate ester groups (SO_3_^−^) on the CNC surface after treatment with sulfuric acid. The increase in surface charge enhanced interactions with PDADMAC.

Figure 3C shows the XRD patterns of CNC, PDADMAC, and PDADMAC-CNC hydrogel. The XRD pattern of CNC displayed characteristic peaks of cellulose as mentioned above. The PDADMAC polymer exhibited an amorphous structure and no significant crystalline phase due to highly flexible polymeric chains, which were suitable to interact with CNC and formed hydrogels. The diffraction peaks at 15.5°, 22.6°, and 34.6° in the XRD pattern of the PDADMAC-CNC hydrogel represent the CNC crystal plane (110), (200), and (004), respectively. Compared with CNC, the XRD peak intensity of the hydrogel was reduced because of the electrostatic interactions with PDADMAC, leading to partial disruption of crystalline order. However, the crystalline structures of CNC have remained largely intact in the hydrogel networks.

The swelling ratio of the PDADMAC-CNC hydrogel in different pH media at 37 °C is shown in Fig. 3D and E. The swelling ratio of all the PDADMAC-CNC hydrogel samples increased significantly in the initial 1 h and reached the equilibrium after 4 h. At pH 6.8 and 7.4, the swelling behavior was not significantly different, suggesting the stability of hydrogel under varying pH environments. The swelling ratio of hydrogels was influenced by several factors, including the network structure, polymer interactions, and water absorption capacity. According to the results, as the CNC content in hydrogel formulation increased, the swelling ratio increased possibly due to enhanced hydrophilicity. The hydroxyl (-OH) groups on the surface of CNC allowed them to be hydrophilic. The CNC content increased, and the overall hydrophilicity of the hydrogel also increased, leading to greater water retention in the polymer network. In addition, the swelling behavior of the hydrogel was affected by the electrostatic interactions between positively charged PDADMAC and negatively charged CNC. At higher CNC content, the electrostatic interactions became prominent, leading to increased charge repulsion within the hydrogel network, causing the polymer chain to expand, resulting in the ability of the hydrogel to swell more effectively.

### In vitro release profile of PDADMAC-CNC hydrogels

The encapsulation efficiency (EE%) and drug loading efficiency (DLE%) of PDADMAC-CNC(a%) are shown in Table 2. The encapsulation efficiency (EE%) and drug loading efficiency (DLE%) increased with the CNC content in hydrogel, suggesting that CNC enhanced the loading capability of PDADMAC-CNC hydrogel through stronger interactions, thereby improving hydrogel stability.

**Table 2.** Encapsulation efficiency (EE%) and drug loading efficiency (DLE%) of PDADMAC-CNC hydrogel

Samples	Encapsulation efficiency (EE%)	Drug loading efficiency (DLE%)
PDADMAC-CNC(1%)	69.29 ± 0.21	7.67 ± 0.61
PDADMAC-CNC(2%)	83.94 ± 0.91	16.15 ± 0.60
PDADMAC-CNC(3%)	82.56 ± 0.53	17.44 ± 0.65
PDADMAC-CNC(4%)	83.04 ± 0.32	16.96 ± 0.30
PDADMAC-CNC(5%)	87.23 ± 0.27	19.78 ± 0.73

**Fig. 4.**
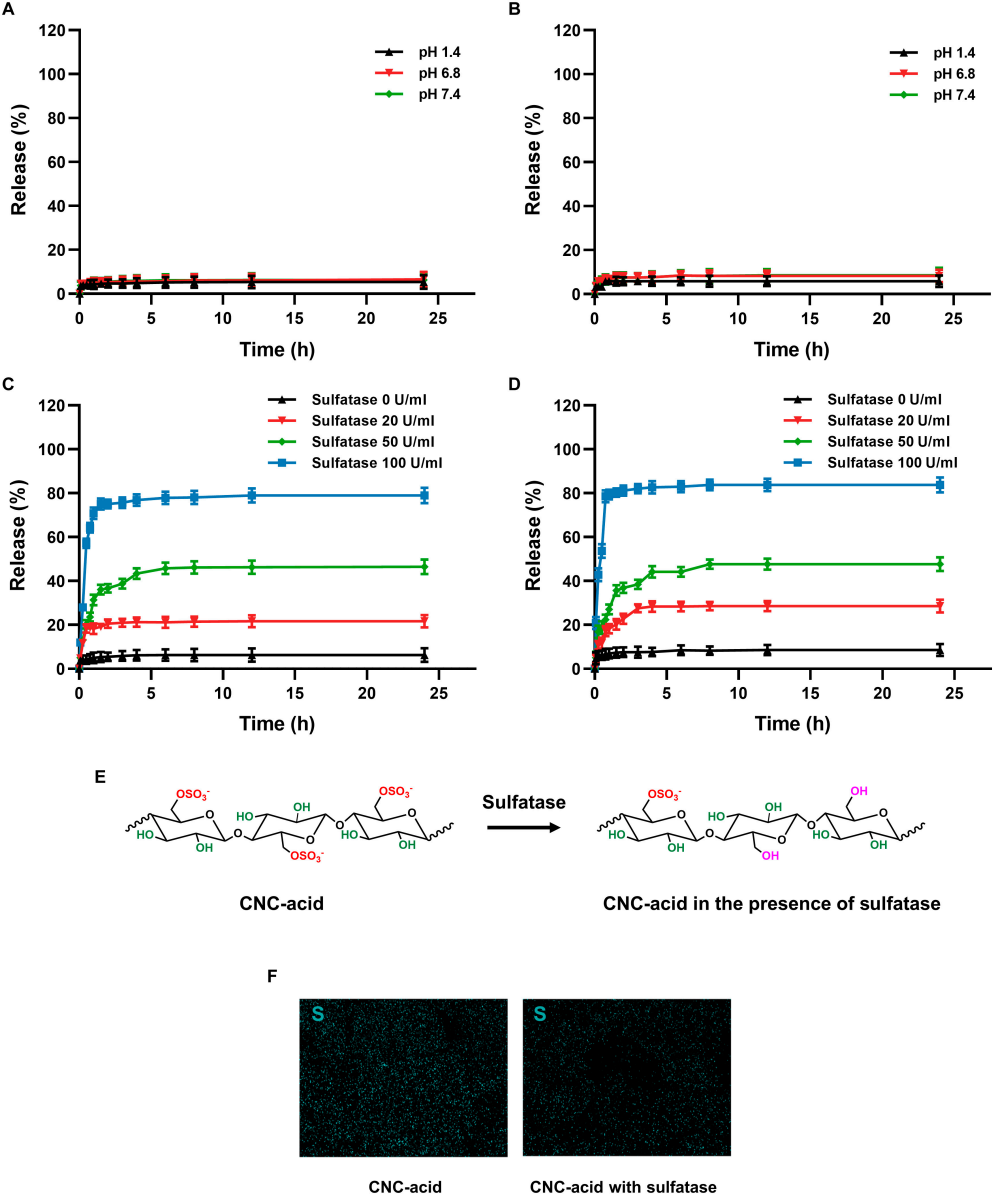
In vitro release profiles of celecoxib drug (A) and DiR (B) at pH 1.4 (SGF), 6.8 (SSIS), and 7.4 (SCS). In vitro release profiles of celecoxib drug (C) and DiR (D) at 0, 20, 50, and 100 U/ml sulfatase. (E) Schematic representation of the hydrolysis of sulfate ester bonds from CNC-acid. (F) SEM-EDS analysis of CNC-acid and CNC-acid with sulfate enzyme.

Figure 4A and B shows the release profile of celecoxib drug and DiR dye from the PDADMAC-CNC(5%) hydrogel at 37 °C under different pH conditions. The release % of celecoxib drug was 5.33%, 6.50%, and 6.25% under SGF (pH 1.4), SSIS (pH 6.8), and SCS (pH 7.4), respectively. The release % of DiR dye was 5.87%, 8.27%, and 8.59% at similar pH values. As a result, the release of payloads from the PDADMAC-CNC hydrogel under different pH conditions showed minimal variation, with the release percentage of approximately 9% indicating that the hydrogel structures remained stable across pH environments. The strong electrostatic interactions between positively charged PDADMAC chains and negatively charged sulfate ester groups CNC created compact stable hydrogel networks. Both PDADMAC and CNC were not pH-dependent interactions; therefore, the hydrogel could maintain network structure, leading to a steady release of celecoxib drug and DiR dye.

The release profile of celecoxib drug and DiR dye from the PDADMAC-CNC hydrogel at 37 °C under sulfatase enzyme is shown in Fig. 4C and D. At SCS (pH 7.4), the maximum release % of celecoxib drug was 6.25% when the sulfatase enzyme was 0 U/ml. As the sulfatase enzyme concentrations were 20, 50, and 100 U/ml, the maximal release % increased to 28.59%, 47.67%, and 83.78%, respectively. Similarly, the release % of DiR dye were 8.42%, 28.59%, 47.64%, and 83.78%. The release of celecoxib and DiR from the PDADMAC-CNC hydrogel increased as the concentration of sulfate enzyme increased. This can be explained by the presence of sulfate ester groups on the CNC. In particular, sulfatase enzymes catalyze the hydrolysis of sulfate ester bonds into hydroxyl (-OH) and sulfate ions (SO_4_^2−^) (Fig. 4E) [32], leading to the degradation of CNC within the hydrogel network. As shown in the FT-IR spectra of CNC-acid with sulfatase (Fig. S2), the signal of S=O stretching (1,029 cm^−1^) disappeared after sulfatase treatment, while the O–H bending (1,640 cm^−1^) exhibited increased intensity. This result indicates that sulfatase catalyzed hydrolysis of sulfate ester bonds, leading to the conversion of sulfate groups into hydroxyl (-OH) groups on the CNC surface. The data confirm that sulfatase effectively removed sulfate groups. Furthermore, the SEM-EDS analysis mapping (Fig. 4F and Fig. S3) revealed a decrease in the sulfur atomic content from 1.87% to 0.51% after sulfatase treatment. This reduction corroborates the enzymatic cleavage of sulfatase and further supports hydrogel degradation and drug release. The enzymatic degradation enhanced hydrogel porosity, promoting drug diffusion and ultimately increasing the release of payloads.

Since the PDADMAC-CNC hydrogel remained structurally stable in different pH conditions, the increase of payload release in the presence of sulfatase enzyme confirmed that the hydrogel could release in response to enzymatic activity rather than pH variations.

### In vitro inflammatory response

**Fig. 5.**
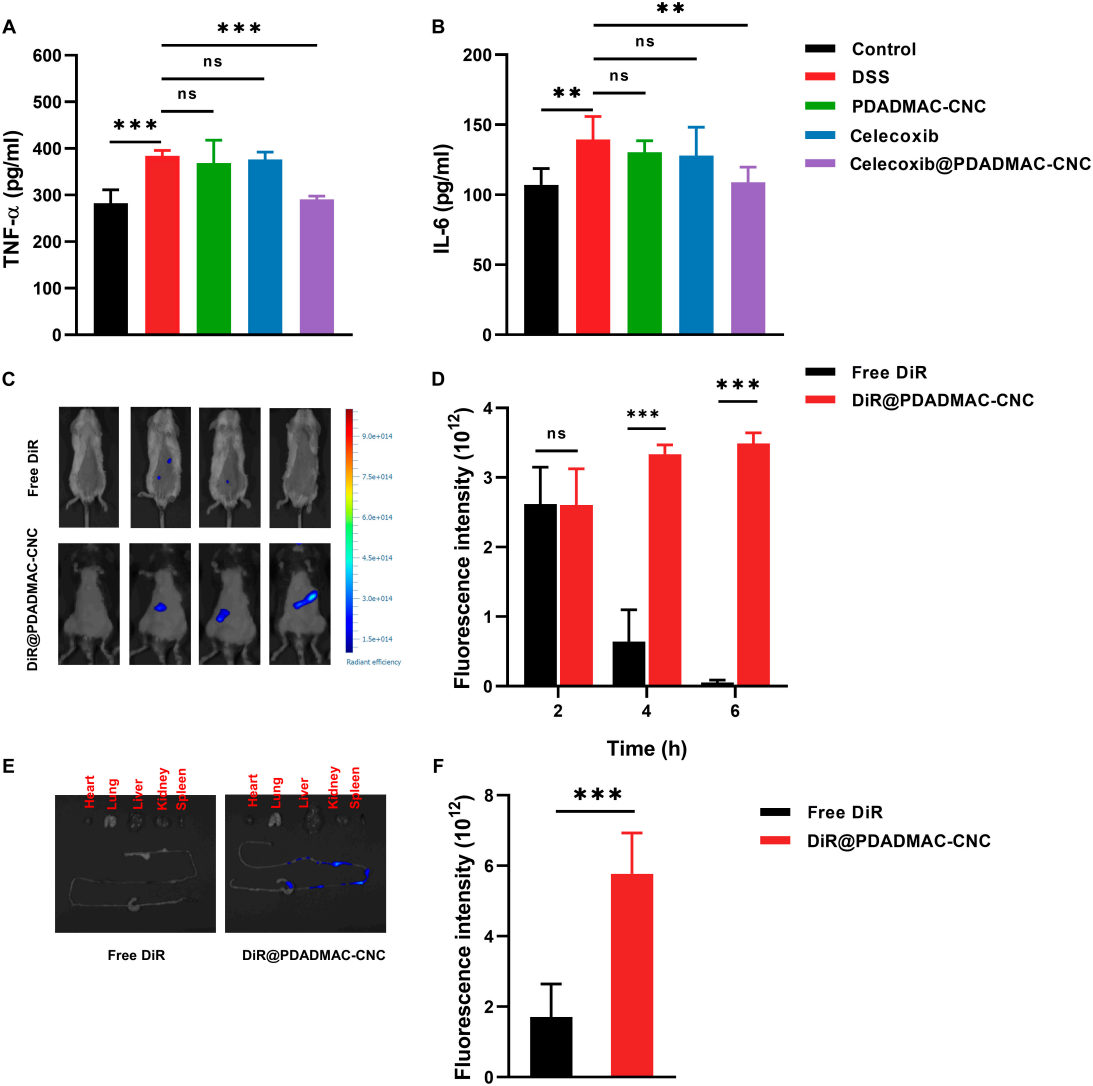
In vitro evaluation of pro-inflammatory cytokine TNF-α (A) and IL-6 (B) levels. In vivo biodistribution images (C) and fluorescence signals (D) of DSS-induced UC mice after orally administered free DiR and DiR@PDADMAC-CNC hydrogel. Ex vivo images of colon and major organs (E) and fluorescence signals (F) 6 h after administration.

Inflammatory cytokines are released by activated macrophages and contribute to the progression of IBD [33]. To evaluate the in vitro anti-inflammatory ability of sample name, the main pro-inflammatory cytokines IL-6 and TNF-α were detected using ELISAs, as shown in Fig. 5A and B. Compared with the control and PBS group, the levels of IL-6 and TNF-α significantly increased after DSS treatment, indicating the successful development of a DSS-induced inflammatory cell model. This symptom was not significantly improved after treatment with the hydrogel or celecoxib alone. As expected, the celecoxib@PDADMAC-CNC hydrogel group significantly inhibited the production of TNF-α and IL-6 and recovered their levels to normal. According to reports, excessive TNF-α could enhance local or systemic inflammation, disrupt the function of tight junctions and intestinal barriers, and lead to the deterioration of colitis. IL-6 is a typical pro-inflammatory cytokine, with high levels involved in exacerbating inflammation and tissue damage [34]. Therefore, the results indicate that the prepared sample has a potent anti-inflammatory effect on DSS-induced inflammation and has the potential to be applied in the treatment of colitis.

### In vivo biodistribution

To evaluate the in vivo biodistribution of the PDADMAC-CNC hydrogel, near-infrared fluorescence dye DiR was used as a model. The fluorescence signals were monitored after the DSS mice were orally administered free DiR and DiR@PDADMAC-CNC hydrogel, as shown in Fig. 5C and D. At 2 h post-administration, free DiR and DiR@PDADMAC-CNC hydrogel exhibited similar fluorescence intensity in the abdominal region, suggesting comparable initial transit through the upper gastrointestinal (GI) tract. However, by 6 h after administration, free DiR fluorescence signals in the abdominal region decreased rapidly, indicating that the dye was systemically absorbed from the GI tract due to its poor stability in the digestive environment. In contrast, mice administered the DiR@PDADMAC-CNC hydrogel exhibited strong and sustained fluorescence, with over a 40-fold increase compared to free DiR, suggesting that the hydrogel effectively prevented dye from degradation and systemic absorption. The prolonged fluorescence retention observed in the DiR@PDADMAC-CNC hydrogel can be attributed to the hydrogel’s stability across the pH conditions of the GI tract, which allows it to maintain structural integrity throughout the stomach and small intestine. This observation was consistent with the in vitro drug release results (Fig. 4A to D) and the strong electrostatic interactions between PDADMAC and CNC, preventing dye diffusion under acidic and neutral conditions. Moreover, at the colon, the sulfatase enzyme could hydrolyze sulfate ester bonds. This process gradually disrupted the hydrogel network, leading to controlled degradation and sustained release of DiR at the target site. Consequently, the fluorescence signal remained localized and strong in the colon over an extended period. These results indicate that the PDADMAC-CNC hydrogel effectively exhibited both pH-dependent stability and enzyme-responsive degradability, enabling controlled release and prolonged dye retention specifically in the colon, thereby demonstrating its potential as an effective platform for colon-targeted delivery.

Moreover, ex vivo fluorescence images of the colon and major organs were observed 6 h after administration to study the biodistribution and retention of the administered samples (Fig. 5E and F). At 6 h post-administration, the DiR@PDADMAC-CNC hydrogel exhibited remarkably higher fluorescence intensity in the intestinal and colon regions compared to free DiR, suggesting that the hydrogel enhanced the stability of dye from degradation and absorption in the upper GI tract. In contrast, minimal fluorescence was observed in the colon following administration of free DiR, suggesting rapid clearance and poor colonic accumulation. Collectively, these results demonstrate that the PDADMAC-CNC hydrogel can effectively protect DiR from degradation and systemic absorption in the upper GI tract, and achieve enzyme-responsive, site-specific release in the colon. This highlights the great potential as a promising platform for colon-targeted drug delivery applications.

### Pharmacodynamics

#### Body weight measurement

**Fig. 6.**
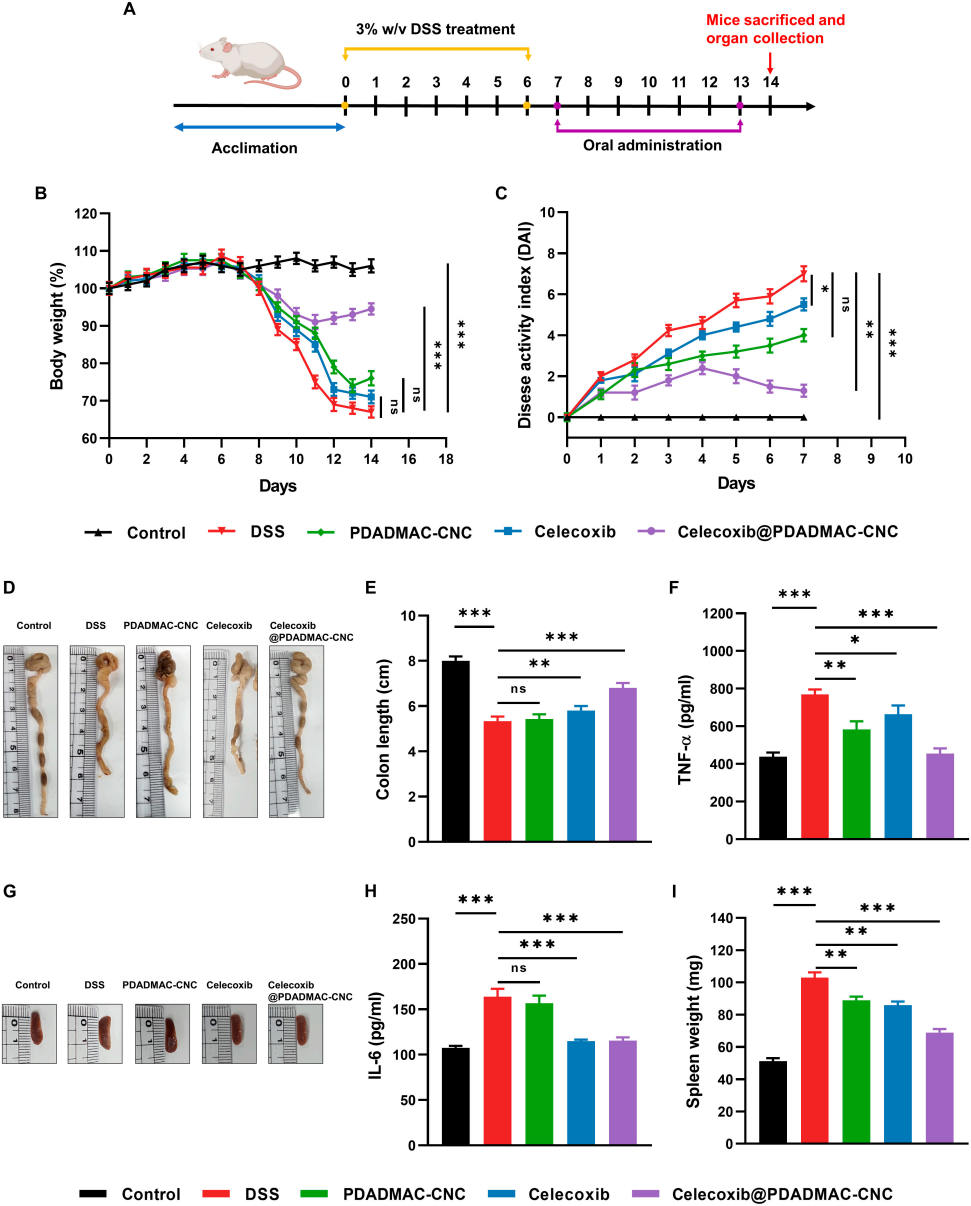
(A) Schematic diagram illustrating administration schedule. Enhanced anti-UC effects on DSS-induced UC mice. (B) Body weight. (C) DAI. Representative colon images (D) and colon length (E) of the mice in each group at day 14. The evaluation of inflammatory cytokine TNF-α (F) and IL-6 (H) levels in the blood of DSS-induced UC mice in each group determined by ELISA. Representative spleen images (G) and spleen weight (I) of the mice in each group at day 14.

To evaluate the effectiveness of the PDADMAC-CNC hydrogel in drug delivery, changes in body weight (%) and the DAI were assessed in mice. As shown in Fig. 6B, all groups maintained stable body weight during the acclimation period. UC was then induced in mice through DSS administration (Fig. 6A). Following treatment administration, mice in the healthy control group (without DSS induction) maintained stable body weight throughout the entire experimental period. In contrast, mice in the DSS group showed a gradual and significant decrease in body weight, reaching 67.10 ± 1.55%, the lowest among all groups. Mice in the DSS group treated with free celecoxib drug showed a slightly higher body weight than the DSS group (71.13 ± 1.70%). The small weight increase in the free celecoxib group was possibly due to the therapeutic efficacy of celecoxib in UC mice. However, the incomplete recovery suggested the side effect of free celecoxib that limited the therapeutic effect in mice [35]. In addition, mice in the DSS group treated with the free PDADMAC-CNC hydrogel exhibited an improvement in body weight of 76.50 ± 1.90%, suggesting that the hydrogel alone may contribute to alleviating inflammation and promoting mucosal healing. Importantly, the body weight (%) of mice in the DSS group treated with the celecoxib@PDADMAC-CNC hydrogel was significantly enhanced to 94.53 ± 1.54% compared to DSS alone. This result suggested the therapeutic efficiency of the drug-loaded hydrogel, which enables targeted and sustained release of celecoxib at the inflamed colon, thereby enhancing therapeutic outcomes while minimizing potential systemic side effects.

#### DAI scores

Figure 6C shows the DAI values, which were used to assess the severity of UC mice. As expected, no signs of inflammation were observed in the healthy control group. In contrast, mice in the DSS group with no treatment exhibited a significantly elevated DAI score (7.23 ± 0.37%), indicating severe inflammation and rectal bleeding, which were characteristic of UC. The DAI values of mice in the DSS group treated with the free PDADMAC-CNC hydrogel resulted in a moderate reduction to 4.50 ± 0.34%, suggesting that the hydrogel may exert an anti-inflammatory effect, potentially due to the physical protection of the mucosa. The DSS mice treated with free celecoxib exhibited a DAI of 5.51 ± 0.35%, indicating a partial therapeutic efficacy in the absence of a delivery carrier. Although celecoxib is intended to reduce inflammation, its high local concentration in a DSS-damaged colon may exacerbate mucosal injury and impair its efficacy, as observed in several studies where celecoxib aggravated colonic ulceration and intestinal damage in mice [36]. Importantly, the DSS mice treated with the celecoxib@PDADMAC-CNC hydrogel group exhibited the lowest DAI score (1.30 ± 0.31%), demonstrating significantly improved therapeutic efficacy. The targeted and sustained release of celecoxib in the inflamed colon enhanced the retention time and improved drug concentration. The sulfate groups on the CNC facilitate interaction with the sulfatase enzyme in the colon, triggering hydrogel degradation and drug release. Moreover, to further assess the severity of colitis among the experimental groups, images of the rectal area were captured from mice in each treatment group (Fig. S4). The images emphasize the rectal bleeding associated with disease activity. Mice in the DSS-induced colitis group exhibited marked rectal bleeding, indicating severe inflammation. Mice treated with free celecoxib and free PDADMAC-CNC hydrogel showed moderate improvement, but the rectal bleeding remained visible. Notably, mice treated with the celecoxib@PDADMAC-CNC hydrogel exhibited no signs of rectal bleeding, indicating enhanced therapeutic efficacy and a marked reduction in disease severity.

#### Colon length analysis

The colon images of mice were studied as an indicator of colonic inflammation in the UC. The shortened and swollen colon reflects the severity of inflammation and tissue damage [37]. As shown in Fig. 6D and E, the colon length of the healthy control was maintained, indicating no signs of inflammation. In contrast, the DSS group exhibited a markedly shortened colon, demonstrating acute inflammation and tissue injury. Similarly, mice treated with the free PDADMAC-CNC hydrogel showed a shortened colon, suggesting the limited effect in the absence of drug loading. Mice treated with free celecoxib demonstrated a modest improvement in the colon length compared to the DSS group, suggesting the anti-inflammatory effect of celecoxib. Importantly, mice in the DSS group treated with the celecoxib@PDADMAC-CNC hydrogel showed a noticeably longer colon compared to the other DSS-treated groups, indicating that the drug-loaded hydrogel effectively reduced inflammation and preserved colonic tissue architecture. The observed recovery in colon length provides the enhanced therapeutic efficacy of the celecoxib-loaded hydrogel. The PDADMAC-CNC hydrogel could protect the celecoxib, enabling the localized release at the inflamed colon, which minimizes the drug degradation in different pH levels of the upper GI tract. The sulfatase-responsive hydrogel could enhance the local concentration of celecoxib at the disease site and minimize systemic exposure. Overall, these results emphasize the potential of the celecoxib@PDADMAC-CNC hydrogel as a highly promising and effective approach for colon-targeted UC therapy.

### Inflammatory cytokine levels in blood

The evaluation of inflammatory cytokines in the blood of DSS-induced UC mice provides critical insight into the severity of systemic inflammatory response and therapeutic efficacy of the treatments [38]. Among the most critical mediators are TNF-α and IL-6, which are elevated in the UC and are well-documented to drive epithelial barrier disruption, immune cell recruitment, and sustained inflammation in UC pathogenesis, contributing to disease progression and tissue damage [39]. As shown in Fig. 6F and G, the DSS group exhibited the highest concentration of TNF-α and IL-6, with the values 769.76 ± 26.02 and 163.76 ± 8.82 pg/ml, respectively, which is consistent with the inflammatory surge reported in acute colitis models [40]. By contrast, the healthy control mice displayed lower concentrations of TNF-α and IL-6, i.e., 438.57 ± 21.62 and 107.63 ± 2.00 pg/ml, respectively. The DSS mice treated with the PDADMAC-CNC hydrogel showed negligible changes in IL-6 but significant changes in TNF-α. In contrast, celecoxib showed significant changes in IL-6 but negligible changes in TNF-α. IL-6 expression is more closely tied to COX-2-mediated prostaglandin E2 (PGE2) signaling in epithelial and immune cells, a pathway that CNC hydrogels do not interfere with in the absence of active drug loading. As a result, IL-6 transcription remains active unless COX-2 is pharmacologically inhibited, such as by celecoxib [41]. TNF-α is rapidly produced by activated macrophages in response to luminal antigens and epithelial barrier disruption, both of which are prominent in DSS colitis. The CNC-based hydrogel, through its biocompatible and mucoadhesive properties, may form a transient protective coating over the damaged mucosa, reducing microbial translocation and dampening Toll-like receptor (TLR)-mediated innate immune activation, thereby suppressing TNF-α release [42,43]. Thus, both PDADMAC-CNC hydrogel and free celecoxib offer partial barrier protection and anti-inflammatory benefits. Notably, the concentration of both TNF-α and IL-6 in the DSS mice treated with the celecoxib@PDADMAC-CNC hydrogel decreased to 454.62 ± 27.41 and 115.53 ± 3.47 pg/ml, respectively, which are in proximate to the levels of control healthy mice. This reduction suggests that the celecoxib@PDADMAC-CNC hydrogel effectively suppressed the inflammatory response. The decreased cytokine levels are attributed to the localized release of celecoxib at the inflamed colon site, enhancing the therapeutic efficacy and reducing systemic exposure. These results support the anti-inflammatory potential of the sulfatase-responsive hydrogel and targeted treatment for UC.

### Spleen weight analysis

Spleen weight is a well-recognized indicator of systemic immune activation in inflammatory models such as DSS-induced colitis (Fig. 6H and I). In this study, visual comparison of spleens across experimental groups revealed substantial differences in spleen weight, correlating with the severity of inflammation and treatment efficacy. As expected, mice treated with DSS alone exhibited marked splenomegaly, a result of increased hematopoietic activity and immune cell proliferation in response to systemic inflammation. Enlarged spleens in DSS mice are consistent with previously reported findings in colitis models, where elevated levels of TNF-α and IL-6 drive splenic immune expansion and lymphoid hyperplasia [44]. In contrast, spleen weight in mice treated with the celecoxib-loaded PDADMAC-CNC hydrogel was notably reduced and approached that of healthy control mice, suggesting effective control of systemic immune activation. This reduction reflects the hydrogel’s ability to deliver celecoxib in a localized, sustained manner, thereby limiting inflammatory cytokine spillover into systemic circulation. Mice treated with free celecoxib or hydrogel alone showed partial normalization of spleen weight, indicating moderate systemic immune suppression, but not as pronounced as the drug-loaded hydrogel group. Collectively, the normalization of spleen weight in the celecoxib@PDADMAC-CNC hydrogel group provides additional evidence supporting its therapeutic efficacy not only at the local (colon) level but also in systemic immune modulation, reinforcing its value in targeted treatment strategies for UC.

### Histopathological assessments

**Fig. 7.**
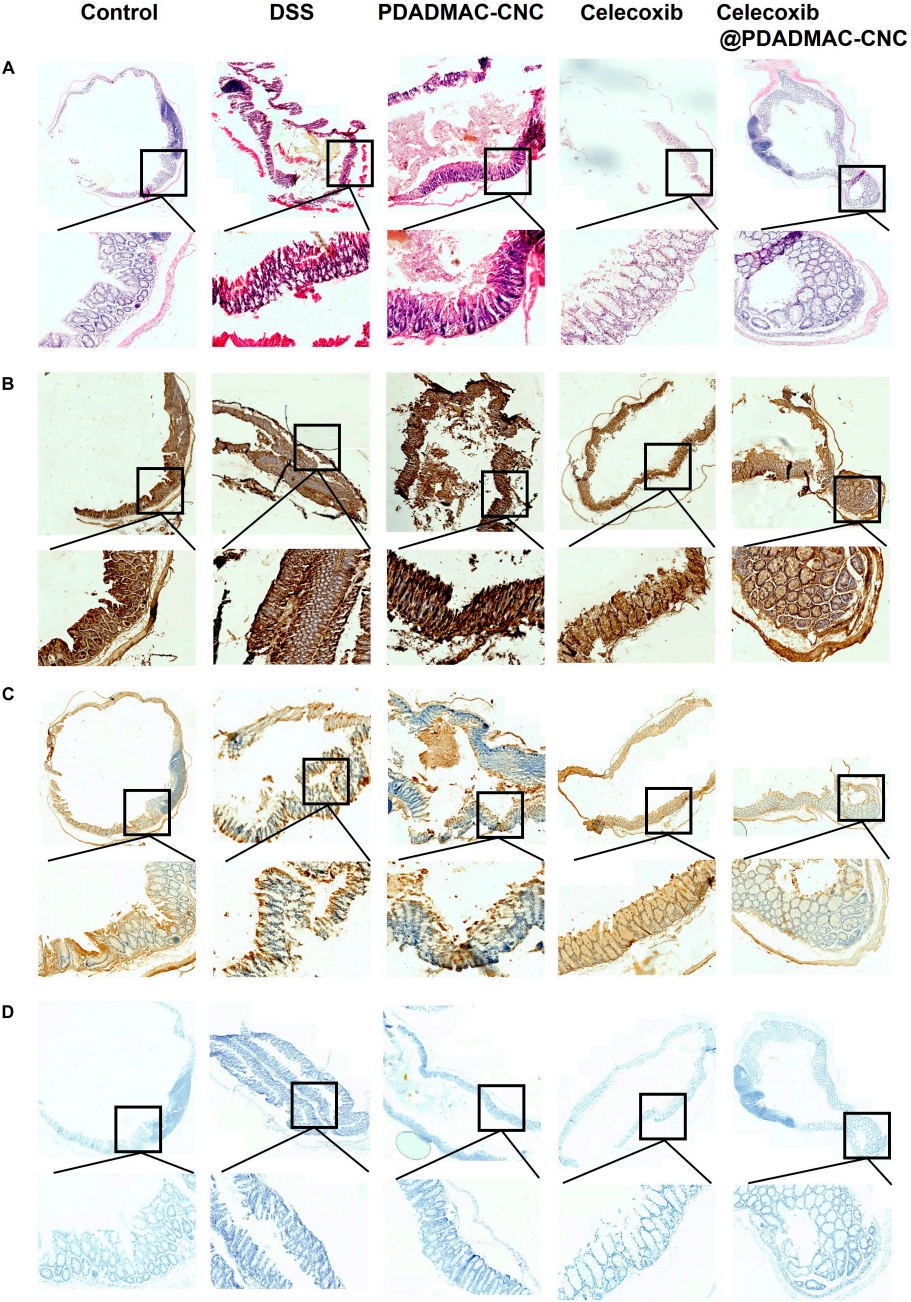
Histological and immunohistochemical evaluation of colon tissues: H&E (A), occludin (B), claudin-5 (C), and ZO-1 (D) staining in each group.

Compared to the intact epithelial structure and well-aligned crypts in the H&E staining of tissue sections from the normal mice, the tissue sections from the DSS mice displayed disrupted mucosal architecture and colonic damage with extensive epithelial erosion, inflammatory infiltration, goblet cell depletion, and loss of crypt integrity, confirming epithelial injury and inflammatory damage. This observation corroborates with the increased inflammation observed in the spleen (Fig. 6H and I) and elevated key pro-inflammatory cytokine levels in the blood of DSS mice (Fig. 7A to D). The H&E staining of tissue sections from DSS mice treated with the PDADMAC-CNC hydrogel showed partial mucosal recovery, crypt formation, and fewer goblet cells, possibly due to the epithelial integrity restoration property of CNC in UC mice, as reported in [45]. However, inflammation and structural damage are still evident in this group, possibly because the cellulose derivatives, such as CNC and cellulose nanofibrils (CNFs), lack anti-inflammatory or immunomodulatory properties [46], which are among the major causes of UC [47]. Furthermore, the H&E staining of DSS mice treated with free celecoxib at 3 mg/kg dose represents large intact crypts and moderate signs of inflammation, as previously reported [48]. However, partially preserved goblet cells, incomplete restoration of epithelial lining, and residual irregularities were observed. In comparison, the H&E staining of DSS mice treated with the celecoxib@PDADMAC-CNC hydrogel showed notably higher restoration of crypt–villus structure, reduced inflammatory cell infiltration, preserved goblet cells, and improved epithelial integrity as indicators of more effective recovery than only celecoxib-treated DSS mice. This is plausibly because the PDADMAC-CNC hydrogel provides protection to celecoxib from the acidic environment of stomach and enables colon-targeted delivery at the site of active inflammation. Moreover, the PDADMAC-CNC hydrogel also prolonged celecoxib delivery, maintaining therapeutic levels (Fig. 4A to D). This suggests that the celecoxib@PDADMAC-CNC hydrogel has enhanced mucosal healing than celecoxib alone in treating DSS-induced colitis.

### Immunohistochemical assessments on the mechanical barrier

Following the histological assessment with H&E staining, which revealed differences in crypt architecture, epithelial integrity, and inflammatory infiltration among treatment groups, immunohistochemical analysis of tight junction proteins (occludin, claudin-5, and ZO-1) was performed to evaluate the functional restoration of the intestinal epithelial barrier. These proteins are essential for maintaining mucosal barrier integrity, and their expression levels and localization provide a more detailed understanding of epithelial recovery beyond structural changes observed in H&E-stained sections. As observed here (Fig. 7A to E), pro-inflammatory cytokines TNF-α and IL-6 are elevated in DSS-induced colitis, which were previously reported to be associated with disrupted epithelial barrier integrity via both transcriptional down-regulation and posttranslational internalization of tight junction proteins [47]. Occludin, ZO-1, and claudin-5 are the key components of tight junctions, which are the structures that seal epithelial cells together, preventing luminal antigens, bacteria, and toxins from translocating into the mucosa [48], making them useful markers for colitis-associated barrier disruption. Compared to the strong and proper localization of occludin (brown-colored DAB-stained cells) and ZO-1 (gray-colored DAB with nickel enhancement-stained cells) at the apical membrane of the colon tissue, the DSS mice showed marked reduction and diffuse occludin and ZO-1 staining. Since occludin is an integral membrane protein that regulates paracellular permeability and ZO-1 is a scaffold protein that links transmembrane tight junction proteins (such as occludin) to the actin cytoskeleton, their loss indicates extensive tight junction breakdown and severe damage to the intestinal epithelium barrier during acute colitis [40]. Furthermore, the claudin-5 expression along epithelium, also shown as brown-colored DAB-stained cells, also indicates normal tight junction integrity in normal healthy mice. In contrast, the expression of claudin-5 in DSS-induced colitis appears fragmented, discontinuous, and mostly cytoplasmic or pericellular, rather than tightly localized linear pattern at the cell–cell borders, as seen in healthy epithelium, confirming tight junction disassembly and increased intestinal permeability and mucosal injury under inflammatory colitis [49]. Further, in accordance with the partial restoration of epithelial lining and crypt–villus structure in hydrogel-treated and only celecoxib-treated DSS mice, the tight junction proteins occludin, ZO-1, and claudin-5 also exhibited partially reestablished, yet still diffused expression patterns, indicating ongoing inflammation despite macroscopic colon tissue healing. Thus, while the PDADMAC-CNC hydrogel and only celecoxib (3 mg/kg) may confer some protective effect against DSS-induced damage, it is not sufficient to fully restore the molecular composition or organization of the tight junction complex. Similar observations have been reported in other study of DSS-induced colitis, where oral administration of celecoxib at 5 mg/kg also showed particle recovery of occludin and ZO-1 proteins [50], and significant recovery of tight junctions proteins in DSS-induced colitis mice at higher doses (10 mg/kg). Here, in this study, it was further observed that oral administration of 3 mg/kg celecoxib loaded in the PDADMAC-CNC hydrogel showed almost complete restoration of occludin, ZO-1, and claudin-5 levels, as evidenced by continuous and intense membrane staining, suggesting enhanced epithelial barrier integrity and improved mucosal healing through effective reassembly of the tight junction complex. These findings highlight the potential of celecoxib@PDADMAC-CNC as a targeted oral delivery system for localized treatment of IBD at doses as low as 3 mg/kg celecoxib.

## Conclusion

In summary, we successfully developed a novel PDADMAC-CNC hydrogel system designed for enzyme-responsive oral delivery of celecoxib to treat UC. The hydrogel exhibited high swelling capacity, favorable biocompatibility, and structural stability under GI conditions. Notably, the presence of CNC in the hydrogel with sulfate ester groups enables responsiveness to sulfatase enzymes, which are abundantly present in the colon. This enzyme responsiveness facilitated localized drug release specifically at the inflamed colon site, thereby improving therapeutic efficacy. In vivo studies confirmed that the celecoxib-loaded PDADMAC-CNC hydrogel effectively reduced colonic inflammation, as evidenced by restored colon length, improved histological scores, and reduced pro-inflammatory cytokine levels in the blood. Moreover, the hydrogel system successfully minimized systemic side effects commonly associated with free celecoxib administration, as supported by improved spleen indices and overall clinical outcomes. Overall, this work highlights the potential of enzyme-responsive hydrogels as a targeted, safe, and effective approach for treating IBDs, such as UC. The developed PDADMAC-CNC hydrogel represents a novel oral delivery system for the treatment of UC.

## Ethical Approval

The animal studies were conducted in accordance with the National Research Council’s Guide for the Care and Use of Laboratory Animals, and animal protocols were approved by the IACUC of Kangwon National University (KW-240715-3), following ARRIVE guidelines.

## Data Availability

The datasets used and analyzed during the current study are available from the corresponding author on reasonable request.
