# Supplementary material for: Cellulose Nanocrystal-Based Sulfatase-Responsive Hydrogel for Sustained Celecoxib Release in Ulcerative Colitis Therapy
Source: Biomater Res. 2026 Feb 3;30:0304. doi: 10.34133/bmr.0304 (PMC12864653; doi:10.34133/bmr.0304)
Supplement: Supplementary 1 — Figs. S1 to S4 [file bmr.0304.f1.docx]

**Supplementary Material**

**Cellulose nanocrystal-based sulfatase-responsive hydrogel for sustained celecoxib release in ulcerative colitis therapy**

Panalee Pomseethong ^a,1^, Mydhili Govindarasu ^a, 1^, Garima Sharma ^b^, Yuyuan Guo ^a^,

Jomon George Joy ^a^, Songrae Kim ^c^, Seung-Hwan Lee ^b, *^, and Jin-Chul Kim ^a, *^

^a^ Department of Biomedical Science & Institute of Bioscience and Biotechnology, Kangwon National University, Chuncheon, 24341, Republic of Korea.

^b^ Institute of Forest Science, Kangwon National University, Chuncheon, 24341, Republic of Korea.

^c^ Metropolitan Seoul Center Korea Basic Science Institute (KBSI) Seoul, 02841, Republic of Korea.

* Corresponding author.

E-mail address: [Ishyhk@kangwon.ac.kr](mailto:Ishyhk@kangwon.ac.kr) (S.-H. Lee) and ([jinkim@kangwon.ac.kr](mailto:jinkim@kangwon.ac.kr) (J.-C. Kim).

^1^ These authors contributed equally to this work.

**
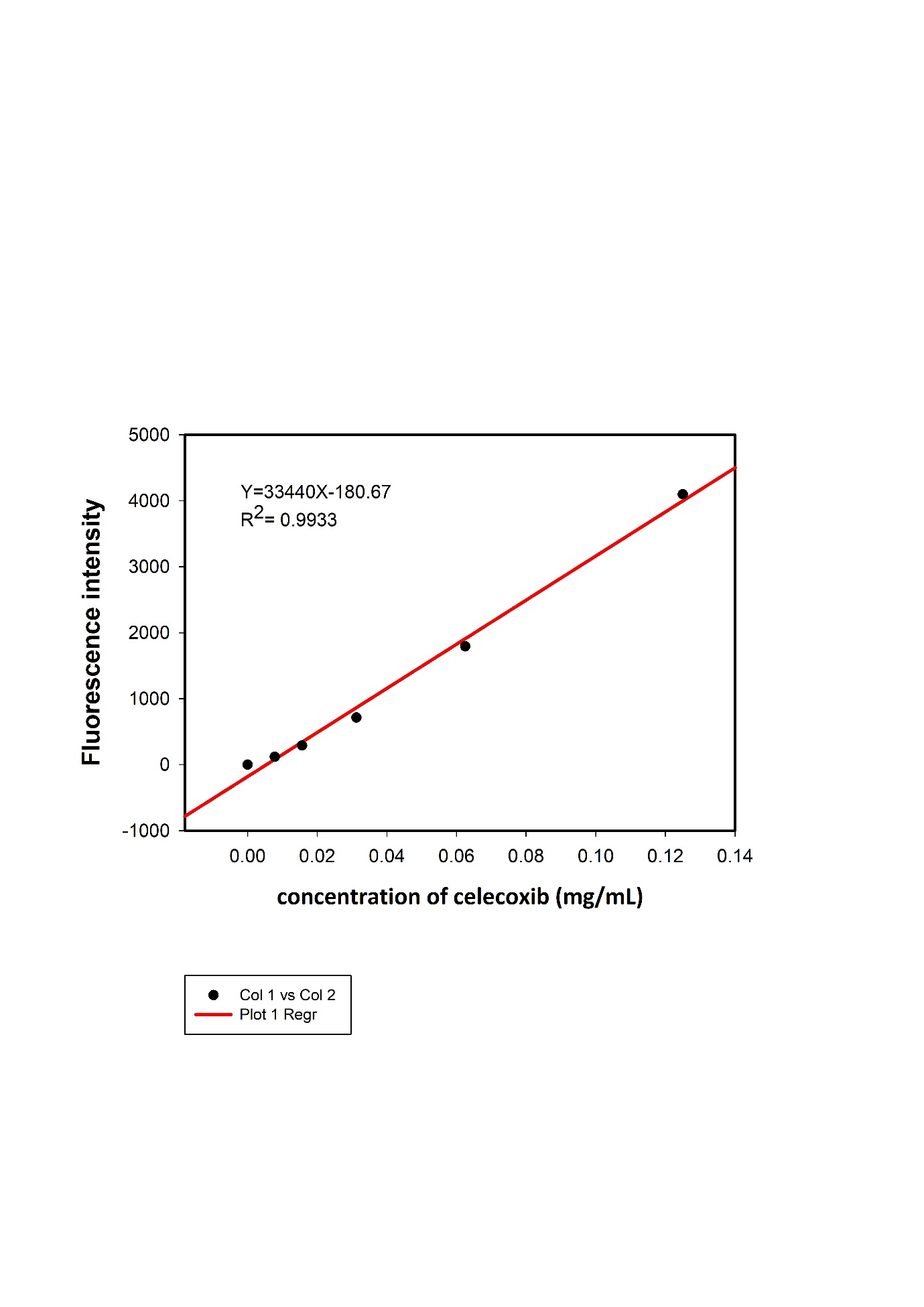
**Supplementary Figures

**Figure S1.** Fluorescence intensity versus concentration of celecoxib standard curve.
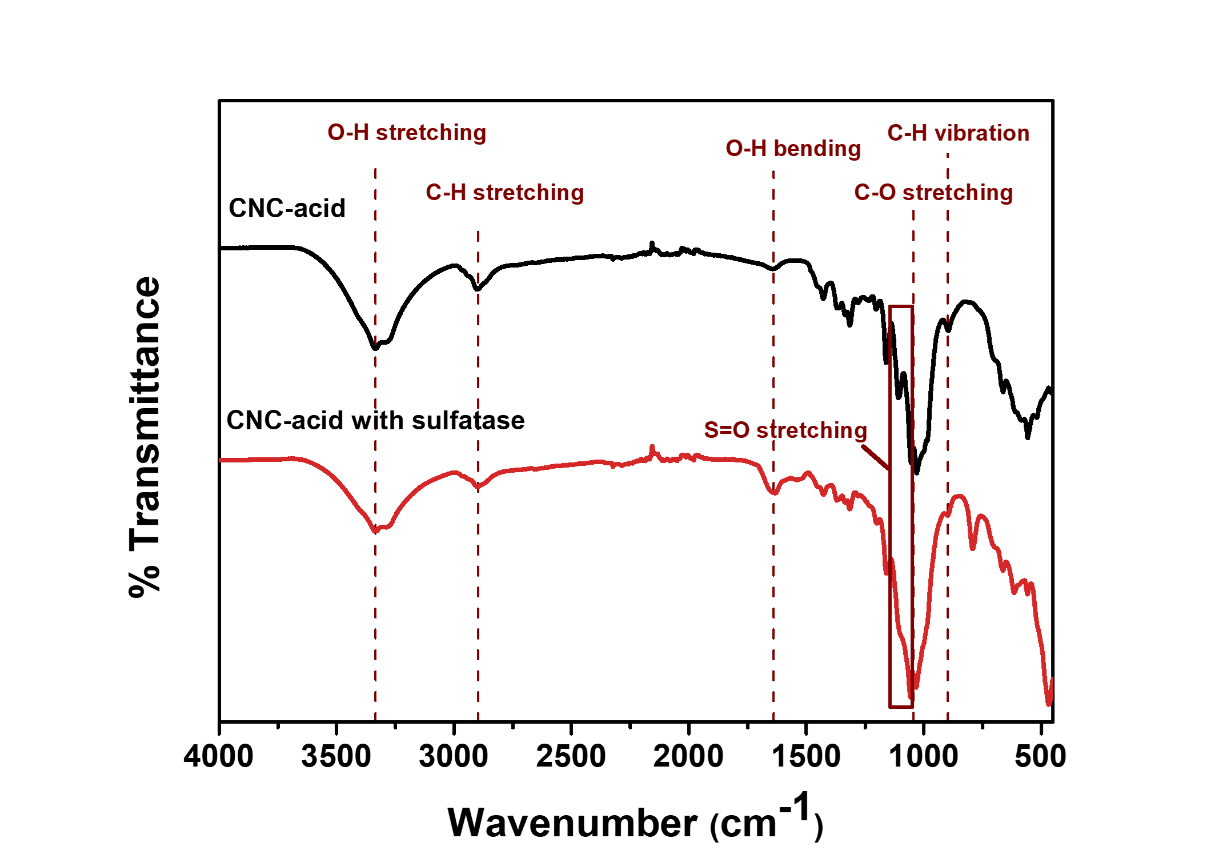


**Figure S2.** Fourier-transform infrared (FT-IR) spectra of CNC-acid and CNC-acid with sulfatase


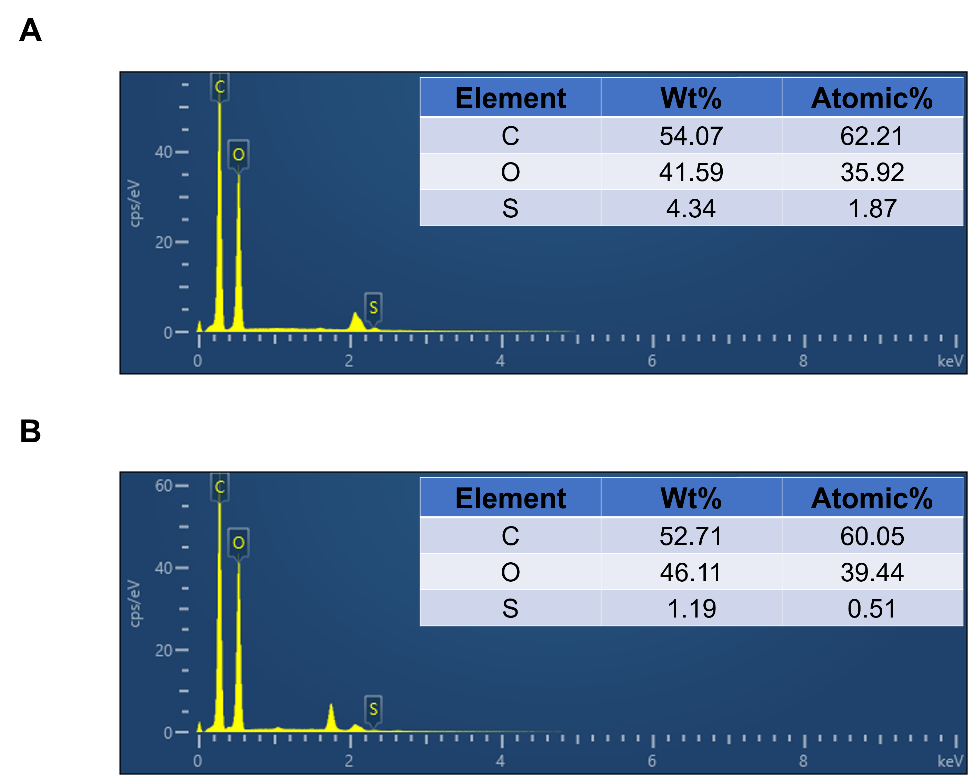


**Figure S3.** SEM-EDS analysis mapping of CNC-acid (A) and CNC-acid with sulfatase (B).

**
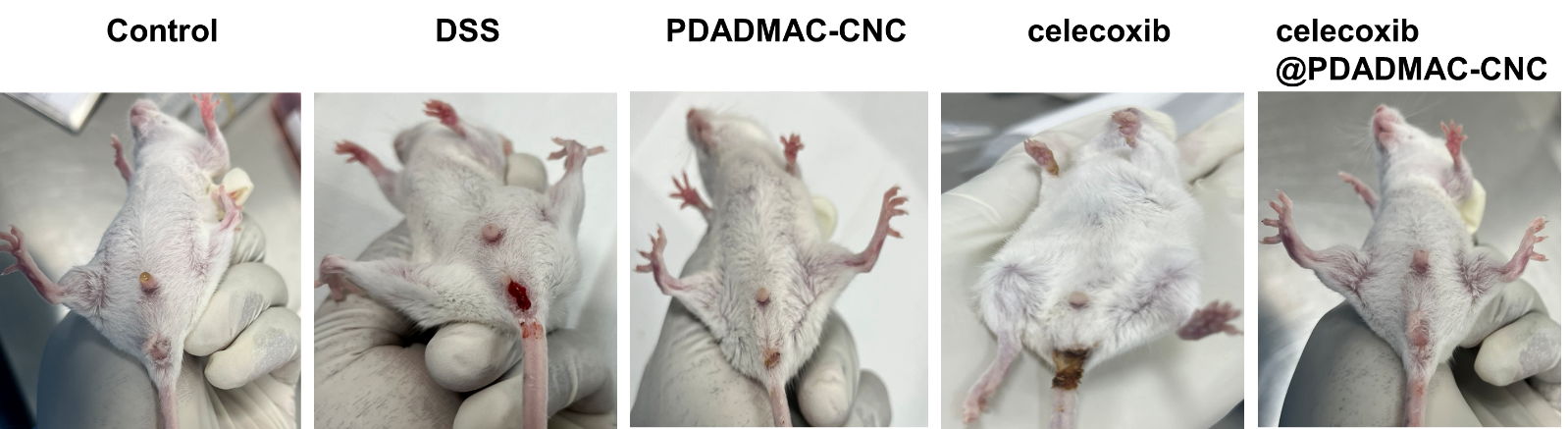
**

**Figure S4.** The images of rectal area from mice in each treatment group.
